# First-degree family history of diabetes and its relationship with serum osteocalcin levels independent of liver fat content in a non-diabetic Chinese cohort

**DOI:** 10.1186/s12889-019-7932-5

**Published:** 2019-12-03

**Authors:** Yiting Xu, Yun Shen, Xiaojing Ma, Chengchen Gu, Yufei Wang, Yuqian Bao

**Affiliations:** Department of Endocrinology and Metabolism, Shanghai Jiao Tong University Affiliated Sixth People’s Hospital; Shanghai Clinical Center for Diabetes; Shanghai Key Clinical Center for Metabolic Disease; Shanghai Diabetes Institute; Shanghai Key Laboratory of Diabetes Mellitus, Shanghai, 200233 China

**Keywords:** First-degree relatives of patients with diabetes, Osteocalcin, Liver fat content, Insulin resistance

## Abstract

**Background:**

First-degree relatives of patients with diabetes (FDR) tend to have impaired insulin activity, which lead to the alternation of circulating cytokine levels. Liver is a main target tissue of insulin action; therefore, liver fat content (LFC) has a close relationship with insulin resistance. This study aimed to find the alteration in serum osteocalcin levels in FDR and the relationship of serum osteocalcin levels with FDR and non-alcoholic fatty liver disease (NAFLD).

**Methods:**

In total, 1206 subjects including 413 men and 793 women from the communities, aged 59.7 (range, 54.8–64.3) years, were enrolled. An electrochemiluminescence immunoassay was performed to measure the levels of serum osteocalcin. LFC was measured using quantitative ultrasonography.

**Results:**

A significant decrease was found in serum osteocalcin levels in subjects with NAFLD (*P* < 0.001) as well as in FDR (19.8 ± 5.7 ng/mL versus 20.7 ± 6.8 ng/mL, *P* = 0.028). Furthermore, among the subjects with NAFLD, those with FDR had lower levels of osteocalcin than those without FDR (*P* = 0.011). The presence of FDR remained a predictor for decreased serum osteocalcin levels after adjusting for body mass index, blood glucose, blood lipids, and LFC (standardized *β* = − 0.057, *P* = 0.028).

**Conclusions:**

FDR had lower serum osteocalcin levels than non-FDR. The inverse association between FDR and serum osteocalcin levels was independent of metabolic factors.

## Background

Genetic defects play an important role in the progression of diabetes in susceptible individuals. A few specific genetic abnormalities predisposed to impaired insulin action in vivo were confirmed in a preliminary study [1]. Accumulating evidence suggests that the genetic background of first-degree relatives of patients with diabetes (FDR) has an effect on insulin resistance, leading to a high risk of metabolic complications such as overweight/obesity, hyperglycaemia, and dyslipidaemia [2, 3]. Moreover, genetic defects in FDR can regulate the transcription of cytokine genes, resulting in abnormalities of the secretion and metabolism of cytokines [4]. Our previous studies have found that the levels of adipokines in FDR are significantly increased, possibly because of impaired insulin function [5].

Bone has recently emerged as an endocrine organ that secretes a relatively abundant non-collagen protein, osteocalcin. This small peptide is primarily produced by osteoblasts and partly released into blood, where its concentration serves as a hormone implicated in the regulation of glucose and lipid metabolism, improving insulin sensitivity and stimulating insulin secretion as well [6–10]. Liver is the main site of insulin resistance. Our previous study reported that subjects with non-alcoholic fatty liver disease (NAFLD) diagnosed by ultrasound had significantly decreased serum osteocalcin levels. In addition, serum osteocalcin levels were closely related to fatty liver index, which was estimated based on laboratory tests [11, 12]. However, quantitative measurement by ultrasonography has higher sensitivity than traditional methods in detecting mild hepatic steatosis. Not only can it accurately quantify liver fat content (LFC) but it can also reduce the interobserver and intraobserver variability, and therefore, its use in clinical practice might help to identify subjects with an increased risk for metabolic diseases [13, 14].

As there is lack of evidence focused on the relationship between FDR and serum osteocalcin levels, the present study was designed to investigate alterations in serum osteocalcin levels in FDR. Ultrasound quantification was applied to detect LFC to evaluate changes in serum osteocalcin levels in FDR under the influence of liver steatosis.

## Methods

### Subjects

This study included non-diabetic individuals from communities in Shanghai, more details could found in our previous study [15]. All subjects underwent questionnaire, physical and biochemical measurement in the study. Those with positive for either hepatitis B surface antigen or anti-hepatitis C virus antibody, excessive alcohol consumption in the past 12 months (men: ≥ 210 g per week; women: ≥ 140 g per week), therapies that would influence the progression of NAFLD and/or osteocalcin levels (e.g., recent fractures, steroids, thyroxine) were excluded from the study; besides, the exclusion also included diabetes or receiving anti-hyperglycaemic therapy, history of cardiovascular disease, malignancy, thyroid dysfunction, and renal dysfunction. Ultimately, 1206 subjects were recruited with a finished questionnaire that gathered information on past and current medical history as well as current use of medications. A family history of diabetes was collected from responses to the systematic self-reported questionnaire. FDR was defined as individuals who have one or more first-degree relatives (parents, siblings, or children) diagnosed as having diabetes [16].

### Anthropometric and biochemical measurements

Anthropometric assessments such as body weight, height, blood pressure and waist circumference (W) were measured based on standard methods as previously described [17].

After an overnight fast of at least 10 h, all subjects received a biochemical examination. A fasting blood sample was collected from each subject, and they also received a 75-g oral glucose tolerance test to measure 2-h plasma glucose (2hPG). The laboratory test indicators were measured according to previous study, and further included liver enzymes [serum alanine aminotransferase (ALT), aspartate aminotransferase (AST), gamma-glutamyl transpeptidase (GGT)], and serum osteocalcin levels. Laboratory testing methods have been described in previous studies [17]. The homeostasis model assessment–insulin resistance (HOMA-IR) index = fasting serum insulin (FINS) (mU/L) × fasting blood glucose (FPG) (mmol/L)/22.5. An electrochemiluminescence immunoassay was used to determine serum osteocalcin levels (Roche Diagnostics, Mannheim, Germany) on a Roche Elecsys 2010 immunoassay analyser, with the intra- and interassay coefficients of variation as 1.2 to 4.0% and 1.7 to 6.5%, respectively [6].

### Quantitative ultrasonography

An abdominal ultrasonographic examination was performed in all subjects by a trained doctor specialized in ultrasonography, who was unaware of the study design and clinical details of the participants. The details of device were reported in previous study [12].

All the instrument settings, including “gain”, “depth”, etc. were calibrated using a tissue-mimicking phantom (model 057; Computerized Imaging Reference Systems, Norfolk, VA). The regions of interest in the captured images under the ultrasound machine were analysed using the image software certified by the National Institute of Health (ImageJ 1.41o, National Institutes of Health, Bethesda, MD, USA) as detailed elsewhere [13]. The LFC was then obtained based on the following equation: 62.592 × liver-kidney echo ratio + 168.076 × liver attenuation coefficient − 27.863 [13]. The subjects were defined as NAFLD if their LFCs by quantitative ultrasonography were ≥ 9.15% [13].

### Definitions

Diabetes was defined as FPG ≥ 7.0 mmol/L and/or 2hPG ≥ 11.1 mmol/L; impaired glucose regulation was diagnosed when 6.1 mmol/L ≤ FPG < 7.0 mmol/L and/or 7.8 mmol/L ≤ 2hPG < 11.1 mmol/L [18]. Menopausal status was defined as continuous 12-month amenorrhea without other medical causes.

### Statistical analyses

Statistical analyses were performed by SPSS version 20.0 (SPSS Inc., Chicago, IL, USA). Data with normally distributed are expressed in terms of the mean ± standard deviation, whereas skewed data are expressed as the median with interquartile range. Numbers with percentages were used to express categorical variables. The differences between the two independent groups for normally distributed data, skewed data and categorical variables were assessed by using *t*-tests, Wilcoxon rank-sum test and chi-square test, respectively. Pearson correlation analysis as well as liner regression analysis was conducted to determine the associations between serum osteocalcin levels with LFC. One-way analysis of variance was used for trend analyses of serum osteocalcin in the FDR and NAFLD groups. Multivariable liner regression analysis was performed to examine the relationship of serum osteocalcin levels and FDR. All reported *P* values were two-tailed, and *P* < 0.05 was considered statistically significant.

## Results

### Characteristics of the study participants

A total of 413 men and 793 women, aged 59.7 (54.8–64.3) years, were enrolled in this study. Overall, serum osteocalcin levels were 20.5 ± 6.6 ng/mL, and the median LFC was 8.2 (6.6–22.2%). The proportions of subjects with a family history of diabetes or NAFLD were 20.6% (249 patients) and 39.7% (479 patients), respectively. As shown in Table 1, the prevalence of impaired glucose regulation was higher in FDR than non-FDR (48.6% versus 41.0%, *P* = 0.030); however, there was no difference in the prevalence of NAFLD between FDR and non-FDR (*P* = 0.873). Serum osteocalcin levels significantly decreased in subjects with NAFLD than in those without (19.7 ± 6.2 ng/mL versus 21.1 ± 6.8 ng/mL, *P* < 0.001).

**Table 1:** Characteristic of the study subjects

Variables	Total (*n* = 1206)	Non-FDR (*n* = 957)	FDR (*n* = 249)
Age (years)	59.7 (54.8–64.3)	59.9 (54.8–64.4)	59.0 (54.0–63.2)
Men, n (%)	413 (34.2)	337 (35.2)	76 (30.5)
BMI (kg/m^2^)	24.0 ± 3.2	24.0 ± 3.2	24.2 ± 3.3
W (cm)	82.0 (77.0–89.0)	82.0 (77.0–88.0)	83.0 (77.0–89.8)
SBP (mmHg)	129.0 (118.0–141.0)	130.0 (119.0–142.0)	124.0 (114.0–137.0) ^**^
DBP (mmHg)	77.0 (70.0–84.0)	77.0 (71.0–85.0)	75.0 (68.0–82.0) ^**^
FPG (mmol/L)	5.7 ± 0.5	5.7 ± 0.5	5.7 ± 0.5
2hPG (mmol/L)	7.0 ± 1.8	7.0 ± 1.7	7.2 ± 1.8^*^
HbA1c (%)	5.6 (5.4–5.9)	5.6 (5.4–5.8)	5.7 (5.5–5.9) ^**^
HOMA-IR	2.2 (1.5–3.2)	2.2 (1.5–3.1)	2.2 (1.5–3.3)
TC (mmol/L)	5.4 (4.8–6.0)	5.4 (4.8–6.0)	5.3 (4.8–6.1)
TG (mmol/L)	1.4 (1.0–1.9)	1.4 (1.0–1.9)	1.4 (1.1–2.0)
HDL-C (mmol/L)	1.4 (1.2–1.7)	1.4 (1.2–1.7)	1.4 (1.2–1.7)
LDL-C (mmol/L)	3.3 (2.8–3.8)	3.3 (2.8–3.8)	3.3 (2.8–3.9)
CRP (mg/L)	0.9 (0.4–1.6)	0.9 (0.4–1.6)	0.9 (0.5–1.7)
ALT (U/L)	18.0 (14.0–24.0)	18.0 (14.0–24.0)	18.0 (14.0–24.0)
AST (U/L)	20.0 (18.0–24.0)	20.0 (18.0–24.0)	20.0 (17.5–24.0)
GGT (U/L)	22.0 (17.0–31.0)	22.0 (17.0–32.0)	22.0 (16.0–30.0)
LFC (%)	8.2 (6.6–22.2)	8.2 (6.6–22.6)	8.4 (6.6–16.2)
NAFLD, n (%)	479 (39.7)	379 (39.6)	100 (40.2)
Impaired glucose regulation, n (%)	513 (42.5)	392 (41.0)	121 (48.6) ^*^

Continuous variables are expressed as means ± standard deviation or medians with interquartile range. Categorical variables are expressed as numbers with percentages

FDR versus non-FDR, ^*^P < 0.05, ^**^P < 0.01

*FDR* first degree relatives of patients with diabetes, *BMI* body mass index, *W* waist circumference, *SBP* systolic blood pressure, *DBP* diastolic blood pressure, *FPG* fasting plasma glucose, *2hPG* 2-h plasma glucose, *HbA1c* glycated hemoglobin A1c, *HOMA-IR* homeostasis model assessment-insulin resistance index, *TC* total cholesterol, *TG* triglyceride, *HDL-C* high-density lipoprotein cholesterol, *LDL-C* low-density lipoprotein cholesterol, *CRP* C-reactive protein, *ALT* alanine aminotransferase, *AST* aspartate aminotransferase, *GGT* gamma-glutamyl transpeptidase, *NAFLD* non-alcoholic fatty liver disease

### Association between LFC and serum osteocalcin levels

Correlation analysis revealed a significant negative relationship between LFC and serum osteocalcin levels (*r* = − 0.114, *P* < 0.001). Linear regression analysis revealed an independent and negative association between LFC and serum osteocalcin levels after adjusting for age, gender, and menopausal status (in women) (standardized *β* = − 0.142, *P* < 0.001). Furthermore, this association remained significant after additional adjustment of glycated haemoglobin A1c (HbA1c), triglyceride (TG), high-density lipoprotein cholesterol (HDL-C), low-density lipoprotein cholesterol (LDL-C), ALT, AST, and GGT (standardized *β* = − 0.059, *P* = 0.042).

### Comparison of serum osteocalcin levels according to the presence of FDR and NAFLD

**Fig. 1:**
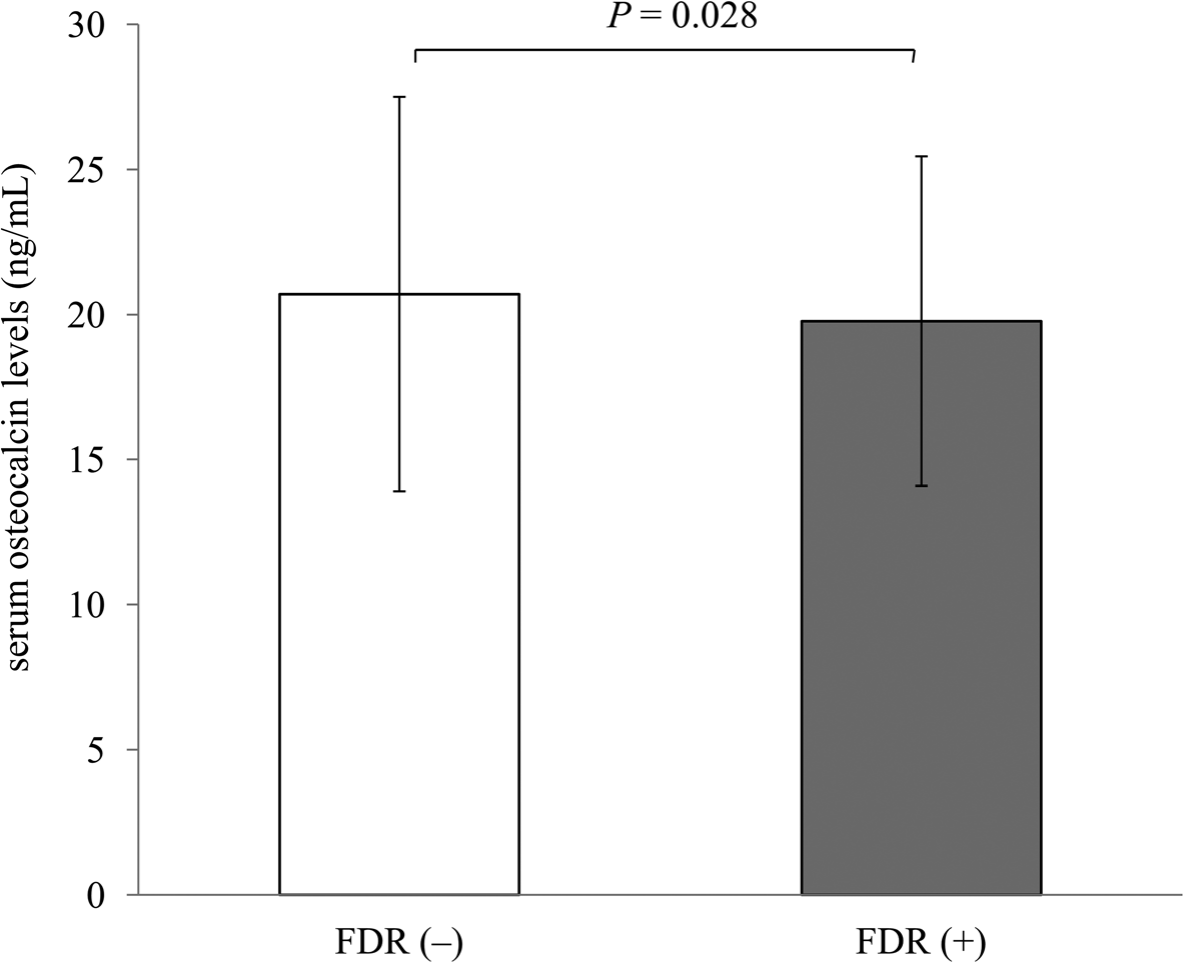
Association of serum osteocalcin levels with FDR. The variables and error bars are shown as mean with standard deviation

**Fig. 2:**
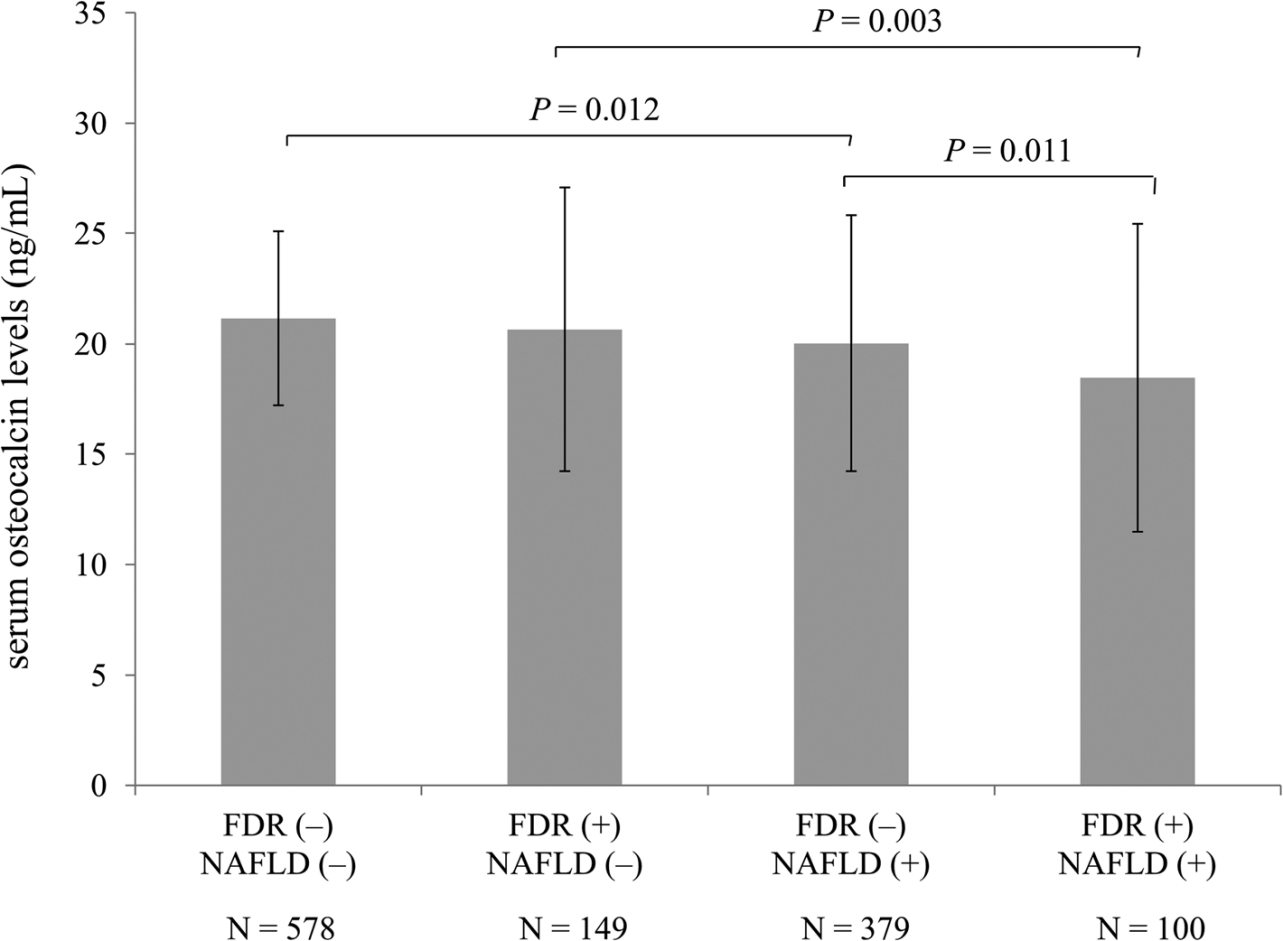
Comparisons of serum osteocalcin levels according to FDR and NAFLD

As shown in Fig. 1, serum osteocalcin levels were significantly lower in FDR than in non-FDR (19.8 ± 5.7 ng/mL versus 20.7 ± 6.8 ng/mL, *P* = 0.028). We further divided the subjects into four groups according to the presence of FDR and NAFLD: group 1, non-FDR + non-NAFLD; group 2, FDR + non-NAFLD; group 3, non-FDR + NAFLD; and group 4, FDR + NAFLD. Figure 2 shows that serum osteocalcin levels were reduced in subjects with NAFLD whether accompanied by FDR or not (*P* = 0.012; *P* = 0.003). In addition, FDR had a lower concentration of serum osteocalcin than non-FDR among subjects with NAFLD (*P* = 0.011). Serum osteocalcin levels showed a decreasing trend from group 1 to group 4 (*P* for trend < 0.001).

### Relationship between serum osteocalcin levels and FDR

A multivariate linear regression analysis was performed to explore the independent relationship between serum osteocalcin levels and FDR. As shown in Table 2, it was found that the presence of FDR was negatively associated with serum osteocalcin levels after adjusting for age, gender, and menopausal status (in women) in model 1 (standardized *β* = − 0.067, *P* = 0.010). In model 2, after further adjusting for BMI, W, systolic blood pressure, diastolic blood pressure, HbA1c, HOMA-IR, TG, HDL-C, LDL-C, and C-reactive protein (CRP), the association remained (standardized *β* = − 0.055, *P* = 0.031). In model 3, the analysis confirmed that after further adjustment of LFC and previous factors, the presence of FDR was an independent determinant of serum osteocalcin levels (standardized *β* = − 0.057, *P* = 0.028).

**Table 2:** Multiple-adjusted associations of FDR and serum osteocalcin levels

FDR	Serum osteocalcin levels		
	standardized *β*	t	*P*
Model 1	−0.067	−2.576	0.010
Model 2	−0.055	−2.154	0.031
Model 3	−0.057	−2.205	0.028

Model 1: adjusted for age, gender and menopause status (in women)

Model 2: adjusted for age, gender, menopause status (in women), BMI, W, SBP, DBP, HbA1c, HOMA-IR, TG, HDL-C, LDL-C, and CRP

Model 3: adjusted for age, gender, menopause status (in women), BMI, W, SBP, DBP, HbA1c, HOMA-IR, TG, HDL-C, LDL-C, CRP and LFC

*FDR* first degree relatives of patients with diabetes, *BMI* body mass index, *W* waist circumference, *SBP* systolic blood pressure, *DBP* diastolic blood pressure, *HbA1c* glycated hemoglobin A1c, *HOMA-IR* homeostasis model assessment-insulin resistance index, *TG* triglyceride, *HDL-C* high-density lipoprotein cholesterol, *LDL-C* low-density lipoprotein cholesterol, *CRP* C-reactive protein, *LFC* liver fat content

## Discussion

In the present study, not only in subjects with NAFLD but also in FDR, the levels of serum osteocalcin went down significantly. Furthermore, FDR exhibited lower serum osteocalcin levels than non-FDR among those with NAFLD. An inverse correlation between serum osteocalcin levels and the presence of FDR remained significant after adjusting for relevant factors and LFC.

In recent studies, bone is recognised as a biologically active organ. Osteocalcin is one of the proteins that play a role in regulating insulin secretion and increasing insulin sensitivity in peripheral tissues. We have found that in both men and women, subjects with serum osteocalcin levels below the median had higher HOMA-IR values compared with those with levels above the median [19]. Moreover, previous studies observed that serum osteocalcin levels were closely related to metabolic diseases due to insulin resistance [17, 20, 21]. The concentrations of serum osteocalcin were significantly lower in NAFLD patients [11, 12]. Lower serum osteocalcin levels were associated with the presence of NAFLD, even in obese people [22]. However, the prediction of LFC in the above studies was based on a model calculated by routine laboratory indicators. Although liver biopsy remains the gold standard for the diagnosis of NAFLD, its value for community-based studies is limited because of its invasive nature. Recent studies using imaging examination not only noninvasively but also accurately estimated LFC; in particular, because of its convenience, ultrasound technology is being developed and applied to research conducted in populations with a large sample. LFC has been involved in some clinical studies related to metabolic abnormalities, atherosclerosis, and cytokines [14]. The present study excluded subjects with diabetes or subjects undergoing lipid-lowering treatments to rule out the influence on serum osteocalcin levels or LFC. We observed a significant negative correlation between serum osteocalcin levels and LFC. Furthermore, using the quantitative cut-off point, it was found that serum osteocalcin levels decreased in subjects with NAFLD, which was consistent with the previous studies. Recent studies have demonstrated a protective effect of osteocalcin against NAFLD, and our previous studies suggested that osteocalcin could improve NAFLD by alleviating oxidative stress and probably by modulating insulin signaling pathway or hepatic lipid metabolism, although the specific underlying mechanisms need to be confirmed with further studies.

In addition, serum osteocalcin levels were also lower in FDR than non-FDR, and the association still existed among subjects with NAFLD. The negative correlation of FDR with serum osteocalcin levels was not influenced by metabolic factors such as BMI, blood glucose, lipids, and LFC. The demonstration of an endocrine feedback loop implied that osteocalcin acted as a hormone improving insulin sensitivity and stimulating insulin secretion; however, its secretion and bioactivity were regulated by insulin signalling. Therefore, the relationship between FDR and serum osteocalcin levels was possibly mediated by insulin resistance.

Ishikawa et al. revealed that FDR tended to be at high risk of developing insulin resistance, and hyperinsulinemia was present even in the absence of obesity [23]. A European multicentre study provided conclusive evidence of the presence of insulin resistance in nondiabetic FDR, even after correction for various covariates [24]. FDR had a defective insulin activation compared with matched controls, and what was worse, insulin sensitivity began to fall from childhood [25]. A study from South Korea also observed a higher prevalence of HOMA-IR in FDR even in the presence of a normal glycaemic profile [26]. Other clinical studies demonstrated that in FDR, insulin resistance, beta-cell dysfunction, and adipocyte dysfunction exhibited altered trends prior to the development of impaired glucose tolerance or diabetes [27].

The potential mechanisms related to the association between FDR and serum osteocalicn levels remain unknown. The presence of insulin resistance in FDR may be an explanation. Wei et al. reported that one molecular mechanism that reduced bone resorption due to insulin resistance leads to decreased insulin signalling in osteoblasts, which caused a reduction in the circulating levels of the bone-derived hormone osteocalcin [28]. The active form of osteocalcin in mice fed a high-fat diet was lower than in the control [28]. Moreover, insulin receptor signalling in osteoblasts controlled osteoblast development and osteocalcin expression. Mice that lacked the insulin receptor had lower circulating osteocalcin levels [29]. Other studies have confirmed the negative effect of insulin resistance on the osteocalcin gene in the osteoblast in vitro [30].

There were several limitations in this study. First, we could not fully elucidate the causal relationship between serum osteocalcin levels and FDR. The role of family history of diabetes in serum osteocalcin levels remains to be explored in further studies. Sceond, the family history of diabetes was self-reported, though accuracy of self-reported family history is highest for first-degree relatives. Third, due to lacking an automated assay to determine under-carboxylated osteocalcin, we only measured its total form, which were proved to be correlated with energy metabolism. In addition, vitamin K was not measured in this study, though circulating osteocalcin levels may be influenced by Vitamin K.

## Conclusion

We found the lower levels of serum osteocalcin in FDR than in non-FDR, which persisted in subjects with NAFLD. The relationship between FDR and serum osteocalcin levels was independent of metabolic factors such as blood glucose, lipids, and LFC.

## Data Availability

The dataset used and analysed during the current study are available from the corresponding author on reasonable request.

## Abbreviations

2hPG: 2-h plasma glucose
ALT: alanine aminotransferase
AST: aspartate aminotransferase
BMI: body mass index
CRP: C-reactive protein
FDR: first degree relatives of patients with diabetes
FINS: fasting serum insulin;
FPG: fasting plasma glucose
GGT: gamma-glutamyl transpeptidase
HbA1c: glycated haemoglobin A1c
HDL-C: high-density lipoprotein cholesterol
HOMA-IR: homeostasis model assessment-insulin resistance index
LDL-C: low-density lipoprotein cholesterol
LFC: liver fat content
NAFLD: non-alcoholic fatty liver disease
TG: triglyceride
W: waist circumference
